# Bio-Based Stabilization of Natural Soil for Rammed Earth Construction: A Review on Mechanical and Water Durability Performance

**DOI:** 10.3390/polym17091170

**Published:** 2025-04-25

**Authors:** Taiwo Sesay, Yuekai Xie, Yue Chen, Jianfeng Xue

**Affiliations:** School of Engineering and Technology, UNSW, Canberra, ACT 2612, Australia; yuekai.xie@unsw.edu.au (Y.X.); yue.chen4@unsw.edu.au (Y.C.); jianfeng.xue@unsw.edu.au (J.X.)

**Keywords:** rammed earth, bio-based materials, mechanical performance, durability, sustainable construction

## Abstract

Rammed earth (RE), despite being an ancient method of construction, has smoothly integrated into contemporary civil engineering due to its compatibility with current sustainability requirements for housing structures. However, typical RE needs some improvements to fully realize its potential as both a structurally effective and environmentally friendly building technique. As a result, multiple bio-inspired enhancement methods have been suggested to substitute traditional cement or lime stabilizers. This review examines the various efforts made in the past decade to biologically stabilize natural soil for the construction of RE. It provides a brief overview of the different bio-based materials utilized in this area but primarily concentrates on their effects on the mechanical strength and water durability of RE structures. The review also addresses current obstacles that prevent the widespread industrial adoption of this valuable earth-building method and identifies potential directions for future research. Overall, the available literature on the mechanical performance and durability of bio-based rammed earth (BRE) shows encouraging outcomes. Nonetheless, various issues, such as the absence of thorough data on the discussed topics, issues related to the inherent properties of soil and biomaterials, and doubts regarding the reliability of durability evaluation methods, have been identified as factors that could lead to a lack of confidence among RE practitioners in adopting bio-based treatments. This study will provide a solid foundation for future researchers aiming to advance BRE technology, thus enhancing sustainability within the construction sector.

## 1. Introduction

With the rapid progress of urbanization, the construction sector faces challenges in fulfilling the growing demands for housing and infrastructure. This in turn creates a ripple effect on the environment, as the production and use of various construction materials such as cement, steel, and aluminum can have significant carbon footprints. To cut down on the already high share of global emissions coming from the physically built environment (about 37%) [1], a transition towards more regenerative material practices by utilizing ethically sourced low-carbon building materials is highly recommended [1]. In the contemporary practice of sustainable construction, the use of raw earth as a primary construction material is becoming prevalent not just with developing countries but also in advanced and industrialized nations such as Germany, France, the United States (USA), the United Kingdom (UK), Australia, and New Zealand. This trend is fueled by the compelling combination of earth’s affordability, availability, and eco-friendliness [2]. Various earth-based construction methods have been employed since ancient times, such as adobe (mud brick), compressed earth blocks, cob, rammed earth, poured earth, and light straw clay. Among these, rammed earth (RE) is arguably the most popular in contemporary housing units, providing numerous advantages, including enhanced indoor air quality, reduced carbon emissions, low consumption of energy and non-renewable resources, cost efficiency, full recyclability, and minimal waste production [3,4].

Rammed earth is a construction method where natural soil, consisting of clay, silt, sand, and gravel, is compacted in layers within a temporary formwork, followed by a drying period to attain strength [5]. Despite the numerous benefits associated with this technique, two primary challenges are typically linked to the natural rammed earth (NRE) mix. The first is that its mechanical performance is often marked by relatively low compressive strength, inadequate tensile and flexural behavior, and limited shear strength [6]. The second challenge pertains to durability, which in this regard refers to the capacity of a structural element to withstand environmental or human-induced wear, damage, or degradation during its anticipated lifespan [7,8]. For earthen units like RE walls, liquid water is considered the most harmful among all environmental factors (e.g., wind, fire, solar exposure, and chemical attacks) [9], as it can infiltrate buildings through various channels such as rainfall, foundation rise, ambient moisture, and utility leaks, significantly diminishing capillary cohesion within the material structure [9]. To resolve these challenges, researchers have implemented various stabilization techniques, including chemical-based additives (cement, lime), industrial by-products (e.g., fly ash, ground granulated blast furnace slag (GGBS), calcium carbide residue, granite sludge, and iron mine spoil waste), recycled concrete aggregate, brick waste, and silica fume [10,11,12,13,14], as well as bio-based materials [15,16,17,18].

The development and incorporation of bio-based materials as soil stabilizers for RE construction have been influenced by their abundant availability in nature, eco-friendliness, renewability, and lower embodied energy compared to their chemical-based alternatives. In general, incorporating biomaterials into earthen architecture is not a novel concept, as historical civilizations are known to have employed organic substances such as animal blood, dung, and plant extracts [19,20]. Nevertheless, extensive research on their application in the RE context only began to be recorded around a decade ago. Previous researchers also conducted literature reviews on the bio-based stabilization of earthen materials featuring multiple construction techniques including earth plaster, cob, adobe, compressed earth blocks, and rammed earth [21,22,23,24]. However, it is challenging to comprehensively review the bio-based stabilization for many earthen construction techniques in a single paper, which may lead to insufficient coverage of various themes such as material properties, mechanical performance, and durability. Furthermore, the types of bio-based materials examined in previous literature reviews on earthen construction were generally limited to either natural fibers (NFs) [14,24,25,26], biopolymers [23], or both [21]. These studies highlight the benefits and possible drawbacks of using NFs and biopolymers in treating earth-based materials. Essentially, NFs are beneficial for enhancing tensile, shear, and flexural strength [14], improving thermal insulation, enhancing ductility, and providing better capacity to balance the air humidity of earth composites [24]. However, the incorporation of NFs into earth-based composites has a limited effect on durability properties such as erosion and abrasion resistance [24]. Additionally, the addition of NFs sometimes decrease compressive strength, except when they are mixed with cement [14]. Conversely, biopolymers are effective at enhancing the compressive strength, stiffness, and erosion resistance of earthen mixtures, although their influence on other mechanical properties like tensile and flexural strength is diminished. Furthermore, due to their organic nature, both NFs and biopolymers are prone to biodegradation, such as mold formation, which poses a significant disadvantage for their application in earthen construction.

Other bio-inspired stabilization techniques, such as microbially induced calcium carbonate precipitation (MICP), have also been reported for RE construction, which was not considered in previous literature reviews. MICP has demonstrated the dual merits of strength efficiency and environmental sustainability in geotechnical applications involving cohesionless soils [27,28,29], proving its significant potential for RE stabilization. This technique is low in energy consumption and relies on natural processes driven by microbial metabolism and biogeochemical reactions to generate calcium carbonate (CaCO_3_). Additionally, the chemicals involved are not linked to carbon-intensive industries like the cement industry. In addition to being environmentally friendly, MICP helps decrease soil permeability, which in turn reduces the risk of liquefaction and enhances water durability performance [30]. However, there are certain limitations that may make MICP less competitive compared to NFs and biopolymers in RE applications. One such limitation is the cost of producing the necessary bacteria and reagents for the cementation solution on a large scale. For instance, the current market price for the patented *S. pasteurii* ATCC^®^ 11859™ is approximately USD 402.0, while the costs of analytical grade yeast extract and urea are roughly estimated at USD 180.0 and USD 111.25 per kilogram, respectively [30]. Other drawbacks of MICP include the production of undesirable NH_3_ by-products from the urea hydrolysis process and challenges in achieving uniform calcite distribution throughout the stabilized soil [31]. Nevertheless, it is our view that global interest in implementing MICP for RE stabilization will increase in the near future, as efforts are encouraged to repurpose NH_3_ by-products as fertilizers and to employ native bacteria and technical-grade reagents instead of the costly analytical grades [31].

Therefore, the present study reviews the published works exclusively related to the use of bio-based materials in NRE (described in Section 2), featuring different bio-treatments, including NFs, biopolymers, and MICP. The study comprehensively reviews their impacts on the mechanical performance and water durability of RE materials. It examines current challenges and potential future research areas to enhance the acceptance of bio-based rammed earth (BRE) technology, thus, contributing its share towards the global efforts of transitioning to lower-emission building materials. Following this introduction, Section 2 describes the literature search methodology. Section 3 presents a brief overview of the different groups of bio-based materials that have been explored for RE applications. The subsequent two sections, i.e., the fourth and fifth sections, are the central themes of this paper, which summarize information regarding the present state of research on how biomaterials influence the mechanical performance and water durability of rammed earth, respectively. Section 6 discusses some challenges faced in adopting the BRE technology and offers some perspectives for future research. Finally, Section 7 gives a general summary and concluding remarks from the study.

## 2. Literature Search Methodology

The studies reviewed in this paper were accessed by conducting a comprehensive search of popular databases, including Google Scholar, Web of Science, and ScienceDirect, using keywords such as ‘rammed earth’, ‘bio-based material’, ‘mechanical performance’, ‘durability’, and ‘sustainable construction’. Other sources, including international reports, website blogs, and national building codes, were also consulted and included in this review. The initial search resulted in more than thirty thousand documents. However, after further conducting a detailed screen of titles, abstracts, and full articles, a total of forty-five scholarly documents (thirty-two research articles, two literature reviews, four conference papers, six book chapters, and one master’s thesis) spanning over the period of 2013–2025 were identified as relevant to this study. These papers/documents were selected based on the following inclusion criteria:

- Only studies about the RE construction method were considered. Research on bio-treatments for other earth construction techniques, such as cob, adobe, poured earth, and compressed earth blocks, was disregarded.
- The literature needed to focus on stabilizing plain RE using bio-based materials or techniques (e.g., NFs, biopolymers, MICP). Studies using bio-based materials as secondary additives in chemically stabilized rammed earth were briefly mentioned but not discussed or analyzed at significant length.
- Emphasis was placed on works investigating the mechanical and water durability performance of BRE mixes. Other performance evaluation themes such as hygrothermal behavior, acoustic performance, thermal comfort, energy performance, and cost evaluation are outside the scope of this review and were therefore not used as a basis for the literature search.

**Figure 1:**
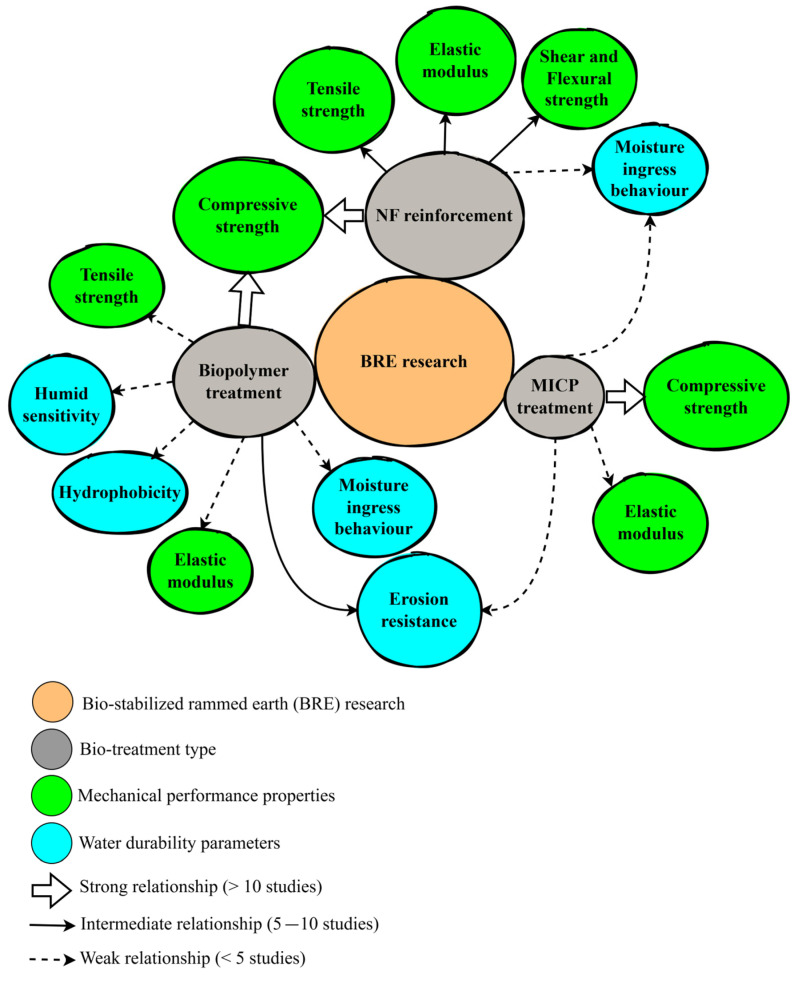
Bubble diagram of literature search showing search areas and key terms.

Figure 1 is a bubble diagram of the literature search showing the search areas and key terms used for search.

## 3. Brief Overview of the Bio-Additive Materials Used in RE Application

**Figure 2:**
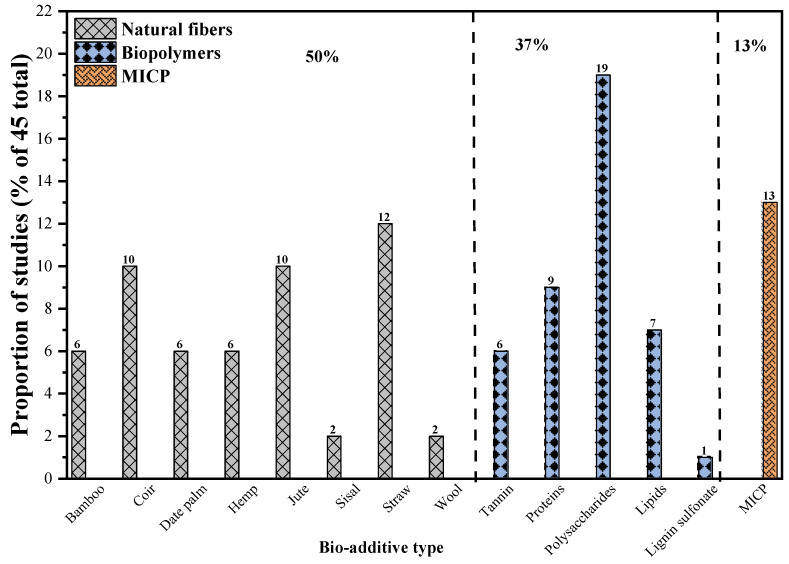
Current research trend on the use of bio-based materials in rammed earth.

Different bio-based materials and techniques have been investigated for their potential application in RE construction, including natural fibers (NFs), biopolymers, and MICP treatment. Figure 2 shows the current research trend on the use of bio-based materials for RE stabilization.

Of the total 45 studies on BRE technology, 50% involved NF-reinforced rammed earth (NFRE), 37% were on biopolymer-stabilized rammed earth (BPRE), and 13% were on microbially indurated rammed earth (MIRE). Figure 2 gives a further break down of the proportion of studies in terms of NF material type, biopolymer type, and MICP investigated, which are discussed in the subsequent sections.

### 3.1. Natural Fibers

One of the earliest considerations about using biomaterials in earthen construction involves using natural fibers [32]. Natural fibers (NFs) are derived from renewable sources, generally plants and animals, and can be extracted without harming the environment. The plant-based fibers include bast (hemp, kenaf, jute, flax), leaf (sisal, palm, abaca), cereal straw (wheat, rice, barley, lavender), rice husk ash, seed (cotton, kapok), fruit (coconut coir, banana, fruit shells), and grass (bamboo, bagasse), while the animal fibers include chicken feathers, wool, silk, and animal hair [22,33,34]. Their incorporation into earthen soil improves mechanical performance [35,36,37,38] and enables a reduction in thermal conductivity but frequently increases water absorption percentages and equilibrium moisture content values [35]. As several studies [39,40,41] have indicated, the performance effects of NFs are strongly affected by their intrinsic physical and mechanical properties as well as the fiber amount used in the stabilization. Table 1 presents the material properties of some different NFs that have been documented to reinforce RE mix. Although not all attributes were reported by the different researchers, these assessments are vital as they can help clarify the influence of NFs on various mechanical and durability properties of rammed earth.

### 3.2. Biopolymers

Biopolymers represent a broader class of biologically derived materials extracted from various sources such as plants, animals, bacteria, algae, and fungi. They possess relevant chemical and physical properties, including hydrophobicity, hydrophilicity, and amphiphilicity, which have been exploited to stabilize raw earth materials. From the literature, biopolymers that have been particularly proposed for RE stabilization can be mainly classified into five distinct groups based on the nature of the molecule responsible for improving cohesion and the physicochemical mechanisms involved. These include polysaccharides, proteins, oils and lipids (fats), tannins, and lignin sulfonate.

#### 3.2.1. Polysaccharides

Polysaccharides are complex carbohydrates composed of chemically distinct monosaccharide monomer units linked together by glycosidic bonds to create linear, branched, and network biopolymers. The frequently encountered glycosidic bonds, including α-1,4-, β-1,4-, and α-1,6-glycosidic, are formed by the loss of a water molecule when two monosaccharide molecules are combined [51]. In the linear chain structure, the structural units are linked together by α-1,4- or β-1,4-glycosidic linkages (such as starch), while in the branched structure, the structural units are linked together by α-1,6 glycosidic bonds (such as cellulose) [52]. Polysaccharides are highly abundant in nature and are currently used commercially across various industries (e.g., food, packing, and tissue engineering). When combined with earthen materials, polysaccharides can form microscopic bonds between the clay layers, enhancing cohesion, improving soil strength, and increasing water resistance [53,54].

As directly applied to RE materials, various polysaccharides, such as xanthan gum (XG), guar gum (GG), chitosan, alginate (ALG), and cellulosic glue (CG), have primarily been researched as potential stabilizers. Among these, XG had received the most attention, which is not surprising since it is the most commercially produced industrial gum. Today, XG is recognized as one of the most interesting biopolymers due to its remarkable structural conformation, physicochemical properties, biocompatibility, nontoxic nature, and wide availability [55]. Following XG, guar gum (GG) is the second most studied polysaccharide for RE stabilization. Recent review studies [56,57] comprehensively dealt with the nature of these two biopolymers, including their chemical structure, surface morphology, and physicochemical properties (viscosity, hydration rate, hydrogen bonding and cation bridging, crosslinking ability) and how they can affect the final properties of soils. In general, both XG and GG can create gel networks that stabilize a soil’s microstructure, thereby improving cohesion, strength, and water durability [58,59,60].

Other polysaccharides, including ALG and chitosan, have also been evaluated for their potential to stabilize RE materials [61,62]. ALG is a class of exopolysaccharides mainly obtained from marine brown algae. They are linear copolymeric blocks of α-(1→4)-linked L-guluronic acid (G) and β-(1→4)-linked D-mannuronic acid residues (M) [63]. ALG has a gel-forming ability [57] which has been explored for its potential to produce a soil-stabilizing effect for various types of earthen structures, including RE [61,64]. Chitosan, on the other hand, is a biopolymer of 2-amino-2-deoxy-(1→4)-β-D-glucopyranose, derived from the alkaline deacetylation of chitin, which is one of the most abundant natural polysaccharides [65]. It had been used to modify the surfaces of materials such as textiles and films, as well as in biomedical applications, transferring its desirable properties of nontoxicity, biocompatibility, and biodegradability [66,67]. In addition, various researchers also investigated the possible geotechnical application of chitosan and reported its potential for soil erosion control and hydraulic conductivity [62,68,69] due to its insolubility in water and hydrophobic properties.

#### 3.2.2. Proteins

A look into the sparse literature on biopolymer-stabilized RE (BPRE) identified studies that dealt with the use of three different protein-based materials, including casein [53,61], animal blood [70], and animal glue [71,72].

Casein, C_31_H_27_NO_4_, is a protein-based biopolymer that makes up 80% of the protein in bovine milk [68]. It is typically a suspension of particles known as ‘casein micelles’, which have both hydrophilic and hydrophobic domains in their primary structure [73]. Capitalizing on these properties, casein can be used to improve the water penetration resistance of earthen soils by providing a surface-coating layer for the soil particles [52]. From an environmental perspective, using casein in RE stabilization offers the possibility of an eco-friendly approach to dealing with the enormous amounts of milk waste disposed of into land and water bodies by dairies every year.

Animal blood has been used in earthen construction since ancient times, and various researchers [5,70,74] have recognized its role in improving strength, moisture resistance, and durability. The composition of blood consists primarily of plasma (which makes up approximately 55%) and solid components [75]. According to Piazza et al. [76], blood plasma and bovine blood have the merit of high clay flocculation activity, which is a desirable property for soil stabilization as it can facilitate the aggregation of some of the clay and silt fractions in a raw earth material into larger framework grains with improved strength [70].

Finally, animal glue (gelatine), which is produced from animal collagen found in bones and tissues, has also demonstrated potential in RE construction by improving the cohesion and durability of soil particles [71]. When mixed with soil, animal glue can create gelatinous structures by forming a network of interconnected protein chains, aiding in the retention of moisture within the soil. This ability to gel slows down the drying process, minimizing crack formation and increasing the strength of earth mix. The protein molecules in gelatine can form hydrogen bonds with clay particles. Furthermore, functional groups such as amine and carboxyl in the gelatine molecules can participate in ionic and covalent bonding with minerals in the clay, thus improving cohesion within the soil mixture.

#### 3.2.3. Lipids

Lipids are the fats and oil components of living beings like plants, animals, and microorganisms. They are energy-rich organic molecules with non-ionic charges and can exist in liquid or non-crystalline solids at room temperatures [77]. Due to their natural hydrophobic property, lipid materials, including linseed oil (LO) and tung oil (TO), have recently been considered as potential bio-based stabilizers for RE materials [53,78]. LO is derived from the flax plant and comprises 90% polyunsaturated fatty acids. These fatty acids are amphiphilic with a polar head and a nonpolar unbranched chain. The oil molecules oxidize and polymerize upon drying by creating a crosslinked network. This phenomenon, called siccativation, is prolonged and can be catalyzed by oxygen, metallic ions, and temperature or light energy [79]. On the other hand, TO is a traditional Chinese vegetable oil extracted from the Tung tree (*Vernicia fordii*). Its most common use is in paints and for the protection of wood chips by providing hydrophobicity even when applied without a drier [80]. In RE application, the inherent water-resistant properties of LO and TO can ensure that they act as a protective layer for RE walls against moisture and other environmental factors [53,78]. This can help mitigate erosion and cracking, increasing the structure’s longevity. Moreover, as these materials are natural and nontoxic, they pose minimal threat to human health and the environment, which aligns well with the ethics of sustainable construction practice. 

#### 3.2.4. Tannins

Tannins (Tan) are complex polyphenols that can be encountered in most plants and have been used since antiquity to make leather from hides, act as a base agent for natural glue production, as natural antioxidants in winemaking, as antifungal, anticorrosion, and antibacterial agents, as well as a dispersant for drilling muds [81,82,83]. In traditional raw earth construction, Tan can be used to strengthen the coatings of earthen walls against moisture attack [84], as they can form chemical bonds with active sites on mineral surfaces [85]. According to researchers who explored the use of Tan in RE stabilization, it is probable that Tan connect individual soil grains and lock particles due to phenolic elements polymerizing and adhering to soil particles [15,53]. This dual action of dispersion through their structure and the ability to connect individual grains of soil particles position Tan as a potential eco-friendly alternative to the traditional chemical stabilization of RE.

#### 3.2.5. Lignin Sulfonate

Lignin sulfonate (LIG) is a derivative of lignin separated from biomass by the sulfite pulping process. It is the second most abundant natural polymer after cellulose, accounting for about 15–40% by weight (wt%) of wood [86]. It contains hydrophilic groups, including phenylic hydroxyl and alcoholic hydroxyl, as well as hydrophobic groups [87]. Although LIG is considered a low-value waste product, it can be used to make high-value products such as syngas, carbon fiber, phenolic compounds, various oxidized products, multifunctional hydrocarbons, and can also act as an additive for concrete and as a component for bricks [88,89,90]. Additionally, several studies in the past have recognized LIG as a promising soil stabilizer for both cohesive and non-cohesive soils, showing improvements in the mechanical and durability performances of the stabilized soil [87,91,92,93]. Some researchers [94,95,96] have claimed that the stabilization mechanism of LIG-treated soils is due to the reduction in the clay’s double layer thickness by the neutralization of surface charges of the clay particles and the subsequent formation of more stable particle clusters or aggregates by LIG polymer binding. In the current literature, only one study was found to have investigated the possibility of using LIG for RE stabilization [15]. The study showed through scanning electron microscopy (SEM) and X-ray diffraction (XRD) analyses that LIG has a pore-filling and surface-coating effect in the RE soil matrix. A similar conclusion was also arrived at by previous researchers [94,95,96,97] who investigated the soil stabilization mechanism of LIG.

### 3.3. Biocementation

Biocementation or microbially induced calcium carbonate precipitation (MICP) is a bio-engineering technique that utilizes the metabolic pathways of bacteria to form calcite (CaCO_3_), a natural cement that binds soil particles together [98]. Calcite formation can occur either through biologically controlled or biologically induced mechanisms, depending on the level of control of the microorganism over the formation process. In the biologically controlled mechanism, the organism can independently synthesize minerals in a form that is unique to the species, regardless of the environmental condition. For instance, three microorganisms, *Bacillus subtilis*, *Sporosarcina pasteurii (S. pasteurii)*, and *Bacillus subtilis* subsp. *subtilis*, were used in the work of Akturk et al. [99] to accomplish the biologically controlled mineralization of CaCO_3_ for RE stabilization. Conversely, the biologically induced process is impacted by the environmental conditions in which the organism exists [100,101]. Due to the variations in bacterial metabolism, there are diverse approaches to achieve MICP, which have been extensively discussed by previous researchers [102]. Among these pathways, hydrolysis of urea (also referred to as ureolysis) is the most straight-forward, productive, controllable, and energy-efficient, making it the preferred approach in the MICP stabilization of rammed earth [17,19,99,103,104]. Additionally, while the metabolic pathways of bacteria are crucial for the MICP process, the method of nutrient delivery and the spatial heterogeneity in calcite distribution are also critical factors in achieving effective MICP treatment. The subsequent sections present a brief overview of the ureolytic activity rate, nutrient delivery methods, and spatial heterogeneity in calcite distribution.

#### 3.3.1. Ureolytic Activity Rate

In the urea-based MICP process, the role of urease is crucial for the hydrolysis of urea, which is affected by the rate of calcium carbonate deposition and the type and concentration of bacteria selected for MICP. Theoretically, using bacterial strains with varying urease activities while maintaining consistent conditions would result in different rates of ureolytic activity and consequently different amounts of CaCO_3_ precipitated over a given period [105]. *Bacillus* and *S. pasteurii* are the primarily utilized bacteria for MICP due to their high ureolytic activity rates. Research shows that *S. pasteurii* displays the highest urease activity when compared to other ureolytic strains under similar growth conditions [105,106,107]. However, alternative studies indicate that specific Bacillus strains may show urease activities that are similar to, or even surpass, those of *S. pasteurii* under particular environmental circumstances. For example, *Bacillus pasteurii*, which has been isolated from soil, is recognized for its fast urea-catalyzing capability, thanks to its strong adaptability to the soil setting. Furthermore, Bacillus megaterium, another Gram-positive strain generally found in soils, has been noted to exhibit urease activity comparable to that of *S. pasteurii,* and it can form endospores that can withstand a wider range of temperatures than those permissible by *S. pasteurii* [108,109]. These findings are beneficial as they present various bacterial strain options that are better suited for different environments, thus validating their application scenarios in MICP-stabilized rammed earth.

#### 3.3.2. Nutrient Delivery Methods

As previously mentioned, a key component of the MICP process is how nutrients are introduced into the soil mix. This significantly influences the final properties of the soil because it affects the yield and distribution of calcite within the soil matrix. Generally, two primary methods are utilized for MICP: the mixing and injection methods, each accompanied by various specific implementation protocols [110]. The mixing method consists of directly combining bacterial or bacterial-reagent solutions with soil particles before the molding of samples. This technique promotes an even distribution of bacteria throughout the soil matrix, which is advantageous for achieving consistent cementation, particularly in rammed earth (RE) soils that exhibit low permeability [110]. On the other hand, the injection method involves pumping bacterial and reagent solutions through the soil sample from one end to another at controlled flow rates or pressures (i.e., through pressurized injection). This method is particularly effective for coarse soils with high permeability and for shallow applications. Variants of this method include one-phase injection, where solutions are mixed before being injected, and staged or two-phase injection, where bacterial and reagent solutions are introduced separately, allowing for retention periods that improve bacterial retention and uniform cementation [111]. Another form of the injection method, called gravimetric injection or surface percolation, uses gravity and capillary forces to introduce solutions into the soil from the surface without the need for specialized injection equipment [111]. When compared to the pressurized injection method, gravimetric injection is more economical and straightforward, especially for shallow or coarse soils. However, its effectiveness can be restricted by the soil’s permeability and the penetration depth [110]. Table 2 provides a summary of research concerning MICP-treated rammed earth, detailing the types of bacterial strains used in the MICP process as well as the nutrient delivery methods, concentrations of the cementation solutions, and application rates in the RE mix.

#### 3.3.3. Spatial Heterogeneity in Calcite Distribution

The distribution of calcium carbonate within bio-cemented soils is generally irregular, as CaCO_3_ often accumulates more in proximity to injection points, causing uneven cementation across the soil matrix [111]. This irregularity appears as variations in crystal size, shape, and concentration, which is a crucial factor affecting the effectiveness of MICP applications [110]. Gaining insight into the underlying causes can facilitate the creation of targeted strategies to achieve consistent cementation, thereby improving soil strength, durability, and overall performance. Attaining a uniform calcite distribution in a RE mix is largely dependent on managing bacterial distribution and the structure of CaCO_3_ crystals [111]. Differences in bacterial activity, reagent flow, and environmental conditions can cause uneven precipitation, leading to heterogeneity [110]. Nevertheless, encapsulating bacteria before soil injection can enhance spatial consistency, as it enables the controlled release and activation of the MICP process, fostering a more uniform distribution of calcite [31]. Ultimately, maintaining steady conditions throughout the treatment process is vital for optimizing calcite uniformity in bio-cemented soils.

To summarize, the above considerations suggest a complex interplay of physical, biological, chemical, and environmental factors that need to be carefully controlled for an effective application of MICP in RE stabilization. As the demand for eco-friendly building methods continues to grow, biocementation through MICP is poised to play a vital role in shaping the future of RE construction, making it a must-explore technology for builders and engineers.

## 4. Mechanical Performance of BRE

### 4.1. Uniaxial Compression Strength

The uniaxial compressive strength (UCS) is an essential mechanical performance metric for rammed earth. Several standards stipulate that for a load-bearing RE structure, the 28-day UCS should be a minimum of 2 MPa [113,114,115]. However, since NRE mix often struggles to meet this requirement, enhancing the UCS has become primary objective for stabilizing rammed earth. The subsequent sections discuss the effectiveness of the different BRE technologies, i.e., NF-reinforced RE (NFRE), biopolymer-stabilized rammed earth (BPRE), and microbially indurated rammed earth (MIRE), in terms of improving the UCS.

#### 4.1.1. NF-Reinforced RE (NFRE)

NFs are usually incorporated into soil mixtures to mitigate issues such as swelling, cracking, and shrinkage, while also enhancing tensile strength and thermal resistance [21,116]. However, recent research suggests that certain NFs can enhance the UCS of RE mixes [15,43,47,49]. In this case, various material properties such as fiber length, fiber diameter, surface roughness, fiber dosage, and the mechanical properties of the NF material (e.g., elastic modulus, tensile strength, and water absorption) were noted in previous studies as having a significant impact on the UCS.

For example, in an experimental study conducted by Raavi and Tripura [43], different amounts of coir fibers (1%, 3%, and 5 wt%) and fiber lengths (25 mm and 50 mm) were investigated for their effectiveness to improve the mechanical performance of rammed earth. It was determined that for optimal UCS performance, the fiber length should be kept under 25 mm and the fiber quantity should not exceed 1 wt%. Similar findings from other research that used different NF materials including sisal fiber [117] and date palm [48] to reinforce the RE mix also indicated an optimum fiber amount of 1 wt%. Giuffrida et al. [117] reported that sisal fiber at 1 wt% dosage can enhance the UCS of the RE by about 33%, even without the addition of secondary additives such as lime and marble saw dust. Bourki et al. [48] tested NFRE samples with different palm fiber contents (0.5, 1, and 1.5%) and found that the 1% palm fiber resulted in the maximum improvement in UCS. Conversely, utilizing excess amounts of natural fiber in a RE mix can have detrimental effects on its UCS. For instance, Mabrouk et al. [46] observed a considerable reduction in UCS when higher volumes of hemp fibers (HF) (25%, 50%, and 75% *v*/*v*) were incorporated into the RE mix. Specifically, RE combined with 25% *v/v* HF experienced a UCS decrease of around 43%, while those with 50% and 75% *v/v* HF reflected reductions of roughly 71% and 90%, respectively. The decline in strength beyond the optimal content (1%) can be attributed to fibers clumping together in various areas of the RE samples. Increasing fiber content within the material leads to a rise in the volume occupied by fibers. Consequently, the material porosity increases, causing “weak areas” to be developed after drying and ultimately lowering the mechanical strength [48]

While the aforementioned studies highlight the importance of limiting the fiber dosage to only small amounts for optimum UCS performance, others have revealed a connection between the physical properties of the NF material including the fiber length, fiber diameter, and surface roughness with its strengthening capabilities. Losini et al. [15] assessed the UCS of RE stabilized with various NFs, including sheep wool fiber (SWF) at 0.25 wt%, citrus pomace (CIT), and grape seed flour (GRA) (which contain some fibers) at 1 wt%. Although the fiber percentages remained below 1 wt%, it was found that only SWF led to a 6.1% increase in UCS, while CIT and GRA fibers resulted in reductions of 32% and 17%, respectively. Losini et al. [15] noted that CIT and GRA fibers are shorter (<2 mm), have a larger diameter (up to 80 microns), and a smoother texture compared to SWF, which may explain why they failed to provide a reinforcement effect similar to SWF in binding the soil grains and enclosing them in the fiber network. By similar indication, Koutous and Hilali [47] highlighted a connection between the mechanical properties of the NFs and their effects on UCS performance. In their study, date palm (DP) and straw fibers were blended in the same weight amount (0.75%) into the RE mix. Although both types of fibers enhanced the UCS of RE, the DP fiber was more effective than the straw fiber, which was attributed to the higher modulus of elasticity, higher tensile strength, and lower water absorption of date palm [24] as compared to the straw fibers [47].

#### 4.1.2. Biopolymer-Stabilized RE (BPRE)

Biopolymers are generally recognized for their ability to strengthen soil materials by promoting interparticle bonding, producing a void filling effect, and inducing surface coating of the soil grains [118]. Due to these favorable properties of biopolymers, various research efforts have been directed towards their potential applications as eco-friendly additives in RE mixtures. Indeed, UCS tests on BPRE samples demonstrated outstanding strength outcomes when polysaccharides such as guar gum (GG) and xanthan gum (XG) were utilized as stabilizing agents, particularly at dosage rates of 1.0 to 3.0 wt% [72,119,120]. Most recently, Abdelaal et al. [72] showed that 1% XG is sufficient to produce a significant reinforcement effect on RE samples, reporting an increase of 3.2 times in the 28-day UCS as compared to the unstabilized rammed earth (URE) sample. The polymeric cementation of XG is characterized by an interparticle bonding that causes cohesion between soil particles, enabling the BPRE mix to withstand high compressive stress and strain prior to failure [121]. Guar gum, on the other hand, was observed by Toufigh and Kianfar [119] to produce a pore-filling effect while enhancing the strength of the RE mix. Muguda et al. [60] further exploited the strength improvement capabilities of XG and GG by crosslinking them in equal amounts of 1 wt%. The combined effect of the two biopolymers resulted in even greater compressive strength compared to their individual use and exhibited performance on par with cement-stabilized rammed earth (CSRE).

Biopolymers derived from lipids and proteins have also been explored for their capacity to enhance the UCS of RE materials. Lipid- and protein-based biopolymers possess distinct hydrophobic coating characteristics that can be particularly beneficial in humid regions with significant soil moisture and suction effects. A recent investigation by Lin et al. [78] assessed the potential of tung oil (TO), a hydrophobic lipid-based biopolymer, as a stabilizer for RE and discovered that TO facilitated notably quicker strength development in RE compared to alternative biopolymers, requiring only 7 days to achieve roughly 85% of its maximum strength. This increase in strength over time was attributed to the clumping of the hardened soil, stemming from the consolidation of the bond between TO and the soil particles [78,122]. A similar mechanism for strength enhancement is believed to also occur in protein-based BPRE that incorporates animal blood and animal glue as biopolymer sources. In the experimental study by Kraus et al. [70], blood-stabilized RE displayed about 36% greater UCS than the untreated control sample after 28 days. This improvement can be explained by the highly active flocculation proteins in bovine blood [76], which aids in the aggregation of the clay and silt fractions within the soil mass, resulting in a more extensive and stable grain structure [70]. Also, animal glue from bovine gelatines enhanced RE soil strength through interparticle bonding effects. To demonstrate this effect, BRE samples containing 1% animal glue were produced and tested for their 28-day compressive strength [71,72]. The results showed that stabilized samples with animal glue achieved higher compressive strengths (up to 6.86 MPa) than URE (1.74 MPa), XG-stabilized samples (5.58 MPa), and samples stabilized with 10% cement (6.36 MPa). Animal glue can form hydrogen, covalent, and ionic bonds with clay particles, resulting in a more interconnected and robust matrix than XG which only involves hydrogen bonding and the physical entanglement of the soil particles [71]

**Figure 3:**
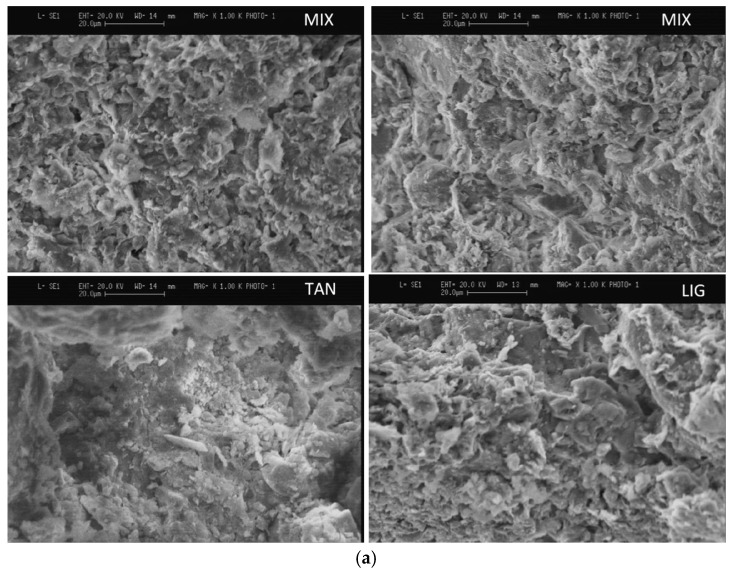

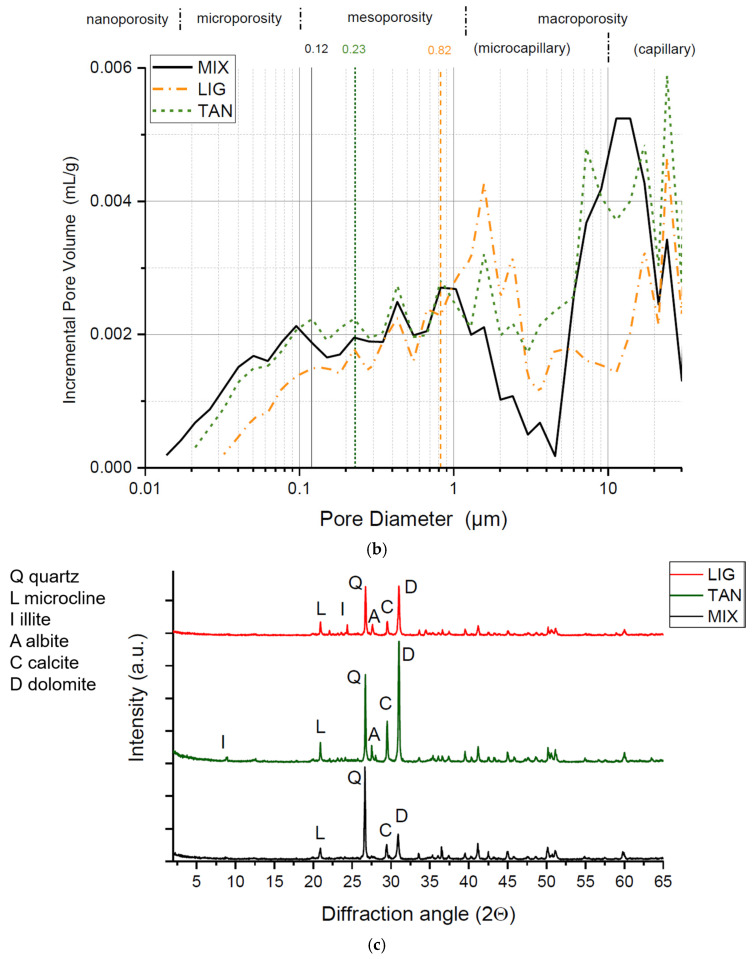
Stabilization mechanism of BPRE mixes based on 1% LIG and TAN biopolymers (reprinted with permission from Losini et al. [15]). (**a**) SEM analysis. (**b**) Pore size distribution from MIP analysis. (**c**) XRD patterns.

As for other biopolymers such as LIG and Tan, the only study that investigated their application as a bio-based stabilizer for rammed earth reported an increase in UCS by about 38% and 13%, respectively [15]. According to Losini et al. [15], both LIG and Tan had a combined effect of pore-filling and interparticle bonding as their strength improvement mechanism. Indeed, several microstructural characterization techniques including SEM, XRD, and Mercury Intrusion Porosimetry (MIP) verified the stabilization mechanism of these biopolymers, as shown in Figure 3.

Upon initial observation of the SEM images in Figure 3a, the URE mix (referred to as MIX) contains smaller particles and aggregates compared to the TAN- and LIG-based mixtures, indicating that the biopolymers contributed to enhancing interparticle aggregation. Similarly, the MIP results presented in Figure 3b show a noticeable change in the pore size distribution of the URE mix following the addition of biopolymer additives. Notably, LIG appears to decrease pore sizes across the meso-, micro-, and nano-ranges, while TAN primarily addresses the reduction of micro- and nanopores. Losini et al. [15] noted that LIG can create a denser and more stable soil structure compared to TAN, while Zhang et al. [123] indicated that raising the LIG concentration from 1% to between 2 and 12% further diminishes the pore volume, even in the macroporosity range. In any event, the MIP analyses demonstrated that the degree of pore-filling effect of biopolymers within a RE soil matrix is influenced by both the type of biopolymer used and the amount used for stabilization. Moreover, the XRD patterns (Figure 3c) illustrated the stabilization mechanisms of biopolymers based on their interactions with the clay fractions in the RE soil. As demonstrated in Figure 3c, the additives did not notably alter the spectra of the MIX soil. However, Losini et al. [15] concluded that the slight decrease in peak intensity of the LIG-based BPRE is due to a surface coating effect of LIG on soil particles, which diminished the intensity of reflection from the incident ray. In general, while the illustrations in Figure 3 pertain to specific BPRE mixes, they provide a representative depiction of the mechanisms by which biopolymers enhance strength in a RE mix.

#### 4.1.3. Microbially Indurated Rammed Earth (MIRE)

**Figure 4:**
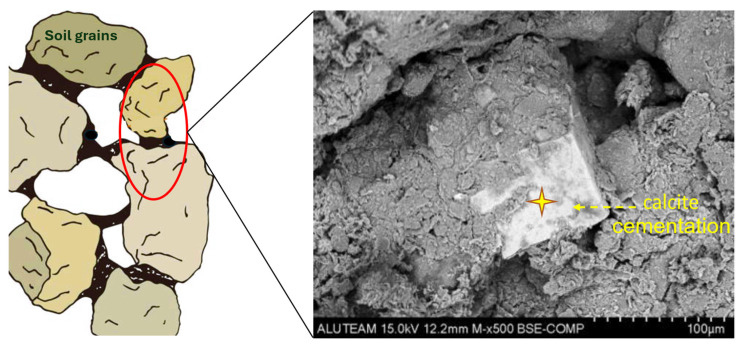
Stabilization mechanism of RE treated with MICP (SEM image reprinted with permission from Aktruk et al. [99]).

MICP is mostly recognized for its role in stabilizing soil, although its use in RE is not as common. However, some researchers have established a positive effect of employing MICP treatment on the compressive strength of RE. Kraus et al. [19] prepared MIRE samples by blending raw earthen soil with a microbial solution containing *S. pasteurii*, calcium chloride, and urea. After a 14-day period, the UCS of the MIRE sample was found to be about 2.5 times greater than that of the URE control. Generally, during MICP, calcium carbonate is deposited in the intergranular spaces of the soil, bonding the grains together, which increases strength [104]. Figure 4 shows a schematic illustration and SEM examination of calcite cementation occurring at the grain-to-grain contacts of a typical MIRE mix.

According to Akturk et al. [99], the strength-enhancing effect of MICP in RE materials strongly depends on the concentrations of microorganisms and calcium in the medium. In their study, the MICP solution did not improve the compressive strength in plain RE mixtures, which was attributed to the lack of Ca^2+^ resources or nutrients for bacteria [99].

As highlighted by previous researchers [124], a significant challenge in employing MICP for soil stabilization is achieving consistent transport, cultivation, and fixation of bacteria. Boling [103] studied optimal methods for cultivating and delivering bacteria to enhance the distribution and quantity of calcite precipitated in the pore spaces of RE materials. The preferred method involved using *S. pasteurii* cultivated in the laboratory and stored as a freeze-dried pellet. In contrast to those maintained as liquid cultures, the bacterial concentrations remained stable over time, allowing MIRE production to commence whenever needed [103]. The source of nitrogen also plays a crucial role in affecting the mechanical performance of MIRE. In the work of Boling [103], MIRE utilizing urea as the nitrogen source demonstrated lower compressive strength compared to URE over different curing durations of 7, 14, and 28 days. However, when pig blood was utilized as the nitrogen source, the UCS of MIRE exceeded that of URE, exhibiting an increase in strength from 280 psi (1.93 MPa) after 7 days of curing to 900 psi (6.21 MPa) after 28 days. Additional compression tests on control RE samples containing blood but no MICP showed that blood was primarily responsible for the observed strength enhancement. While this research was limited to a pilot study, the positive results can provide a basis for future investigations aimed at fully understanding the impact of blood on the compressive strength of MIRE and optimizing the blood concentration in the MIRE formulation.

Overall, the existing research on MIRE technology is largely under-developed. Future research should prioritize establishing optimal conditions for producing MIRE mixes with different bacterial strains. In particular, the optimum bacteria concentration in cementation solution, optimum pH range for MICP, and optimum MICP application rate for RE stabilization need to be clearly defined.

### 4.2. Tensile Strength

Natural fibers, especially those of plant origin (hemp, barley straw, jute, date palm, coconut coir), are often the preferred choice for improving the tensile strength of RE due to their ability to significantly increase the peak strength, reduce the post-crack strength, and change the brittle behavior of earthen materials to a more ductile one [43]. Fiber characteristics, including the fiber length, fiber amount, and the fiber’s capacity to form a strong bond and interfacial adhesion with the soil particles, all play a significant role in determining the tensile response of NFRE. Raavi and Tripura [43] added coconut coir fiber in different fiber amounts (1% to 5 wt%) and lengths (25–50 mm) into URE and CSRE mix and found that the tensile strength improved with an increase in fiber length and amount. This trend was also noted by Hallal et al. [45], who found that the tensile strengths of URE and CSRE enhanced as the amount of HF increased (0.75–1.25 wt%). Conversely, minimal amounts of fiber (<0.5 wt%) in the RE mix may not lead to notable improvements in tensile strength. For instance, Sabbà et al. [49] used a low proportion of WS fiber (0.25%) to stabilize plain RE soil, resulting in a tensile strength of 0.24 MPa, which is lower compared to typical values of 0.4 to 1.0 MPa reported in other studies [45,47]. Koutous and Hilali [47] conducted splitting tensile strength tests on NFRE using BS (0.75 wt%) and DP fibers (0.75 wt%). They observed that although BS has relatively lower mechanical properties [24], it was more effective in improving the tensile strength of RE than DP fiber. BS increased the tensile strength of URE by 31.6%, while DP only improved it by 21.1%, which was attributed to BS’s stronger adhesion with the earthen materials than DP.

Previous studies have also indicated that biopolymers can enhance the tensile strength of RE depending on the amount of additive used for stabilization and their inherent chemical and physical properties [60,119,120]. Notably, XG and GG were examined for their effects on the tensile strength of RE [60,119,120]. Toufigh and Kianfar [119] reported only a marginal increase in the tensile strength of RE stabilized with 2.5% GG (from 0.24 MPa for URE to 0.27 MPa for GG-treated RE) after 28-day curing. Muguda et al. [120] demonstrated that GG enhances the tensile strength of RE after 7 days, but declines over time as the material dries, whereas XG consistently boosts the tensile strength even with extended curing time. This sustained increase in XG over time is attributed to its anionic nature, which forms both ionic and hydrogen bonds with soil particles, leading to improved aggregation and increased suction, thereby enhancing strength. In contrast, GG is a neutral polysaccharide that can create a hydrogel network among soil particles through weak hydrogen bonds [125]. After 7 days of curing, these hydrogels predominantly exhibit rubbery elasticity and contribute to higher suction and tensile strength. However, upon transitioning to a glassy state after 7 days, the hydrogen bonds weaken, and the substantial water retained by the biopolymer chains escapes from the soil mix, resulting in decreased suction and a noticeable reduction in tensile strength [59]. Muguda et al. [60] also showed that the tensile properties of RE can be significantly enhanced when GG is crosslinked with XG at a 1 wt% ratio. The BPRE mixture, after being cured for 28 days, exhibited a tensile strength even greater than that of 8.0–cement-stabilized rammed earth (CSRE) [60]. The crosslinking of these biopolymers appeared to rectify their limitations, offering a chance to modify the stabilization of RE with biopolymers in ways that are not typically achievable with traditional stabilizers like cement.

### 4.3. Elastic Modulus

**Figure 5:**
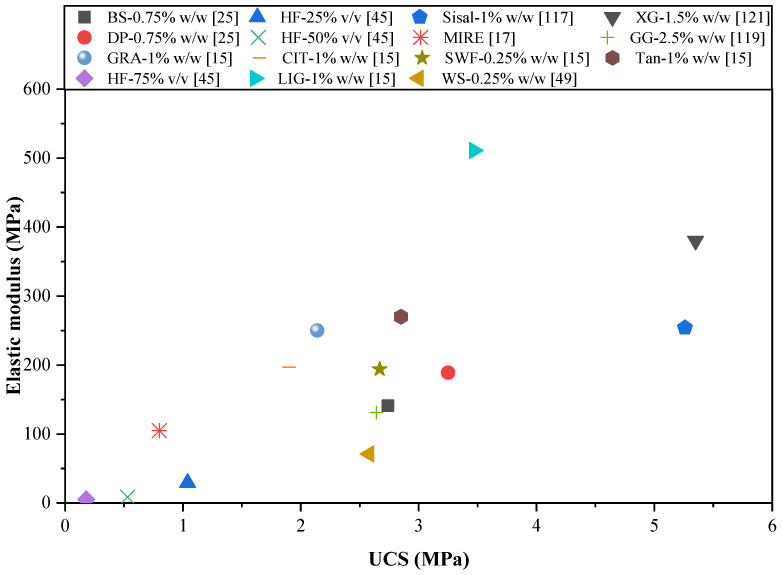
EM—UCS results of different BRE mixtures.

The elastic modulus (EM) is a crucial parameter of RE that defines its elastoplastic mechanical properties. Typically, the EM is derived from the stress–strain curve using UCS data. Nevertheless, there is still no established procedure for its assessment, which complicates direct comparisons of results across the literature. Losini et al. [15] determined the EM of various BRE mixtures as the slope of the chord that runs through one-third and two-thirds of the peak load. Mabrouk et al. [46] focused on calculating the slope of the stress–strain curve within its linear elastic region, while Sabbà et al. [49] performed their evaluations using stress–strain data at 5% and 30% of the maximum stress. Other researchers, such as Koutous and Hilali [47], Patwa et al. [121], Giuffrida et al. [117], and Porter et al. [17], suggested employing the secant modulus (i.e., the ratio of maximum stress to corresponding peak strain) to characterize the elastoplastic mechanical behavior of NFRE, XG-stabilized RE, and MIRE, respectively. Toufigh and Kianfar [119] calculated the EM for GG-stabilized RE by determining the slope of the stress–strain curve, considering the stress at 40% of the ultimate load and the stress that corresponds to a longitudinal strain of 0.000050. It is important to recognize that earthen materials demonstrate a non-linear and inelastic behavior, which implies that any standardized method for calculating the EM can only be utilized to compare various samples and assess the material’s stiffness, rather than to establish the stress–strain relationship. Similar to URE, CSRE, and other earthen building methods, BRE mixtures that exhibit higher UCS values are anticipated to have greater EM values compared to those with lower UCS. However, when analyzing the findings from different studies reporting these two parameters, this trend is not particularly evident, as illustrated in Figure 5. As a result, proposing empirical EM-UCS correlations for earthen materials has faced criticism from some researchers [126], who pointed out that these correlations often stem from inaccurate strain measurements obtained from the actuator of the testing machine, potentially resulting in unrealistic estimations. This highlights the necessity for thorough and comprehensive research to establish a dependable standard procedure for strain measurement and the mathematical evaluation of the EM of BRE mixtures.

As shown in Figure 5, there is a noticeable variation in the EM-UCS data plot for various BRE mixtures. The NFRE mixes yielded the lowest EM values, with HF resulting in measurements as low as 5.1–29.2 MPa, followed by WS and BS fibers. RE mixtures that incorporated DP, SWF, and CIT exhibited comparable EM levels despite displaying significantly different UCS values. In contrast, the biopolymer materials, such as LIG, XG, and Tan, were more effective in enhancing the EM of RE compared to the NF materials. Mixtures based on LIG were observed to achieve an EM of approximately 511 MPa, which is around twice that of the mixtures containing Tan. It is essential to take into account the density factor, which plays a crucial role in influencing the EM, as the various bio-additive materials change the pore structure of the BRE mix [15,46,47]. As noted by Losini et al. [15], LIG created a considerably denser BRE mix in comparison to XG and Tan, effectively reducing the macroporosity of the specimen, which led to its significant positive impact on the EM. Conversely, Mabrouk et al. [46] indicated that the lower EM values in HF-reinforced RE were attributed to the use of the lightweight HF material, which resulted in a less dense mix.

Table 3 provides a summary of the experimental findings concerning the mechanical parameters of BRE derived from various studies.

As indicated in Table 3, the mechanical property that has been most frequently assessed is compressive strength, whereas only a limited number of studies have evaluated tensile strength. Some of the research also reported on the parameters of elastic modulus and ultimate strain. Table 3 presents a broad spectrum of reported values in the literature for the compressive strength and elastic modulus of BRE, ranging from 0.6 to 6.86 MPa and 29.2 to 511 MPa, respectively. All the biopolymers studied consistently demonstrate an enhancement effect (ranging from approximately 13.3% to over 100%) when compared to URE. On the other hand, the findings for NFRE and MIRE show variability. Furthermore, although the tensile strength values for BRE are relatively low (between 0.1 and 0.5 MPa), all related studies indicate an improvement rate from 12.5% to more than 100% higher than URE. Additionally, with a few exceptions, the T/C ratio for the various BRE mixtures falls within the range of 0.07–0.11 as recommended by Bui et al. [128] for RE materials.

### 4.4. Flexural and Shear Strength

Like tensile strength, flexural and shear strength properties are frequently overlooked in the design of RE, as they tend to be quite low [129,130]. However, these properties are crucial for understanding material failure, particularly under dynamic conditions such as wind, waves, traffic, and seismic events. For example, a post-earthquake assessment of RE houses in Tripura, India, revealed that the primary modes of failure include vertical cracks in walls, oblique or diagonal cracks near openings, in-plane (parallel to the direction of force) and out-of-plane (perpendicular to the direction of force) wall collapses, and failures at joints [131]. Addressing or minimizing these failure modes in RE through bio-based additives has been the focus of numerous researchers studying various NF materials, such as coconut coir [42,44], HF [45], jute fiber [16,42], rice straw [16], and bamboo fibers [42,44,132].

Tripura et al. [44] assessed the in-plane and out-of-plane flexural strengths of URE and CSRE wallets that incorporated coconut fibers and bamboo splints. Their findings (0.54–2.11 MPa) significantly exceeded the 0.1 MPa benchmark established in NZS 4297 [129]. Hallal et al. [45] examined the modulus of rupture (flexural strength) of RE enhanced with varying amounts of HF and discovered that the HF-reinforced RE demonstrated at least a 70% increase in flexural strength when compared to the URE control. Similarly, when jute fibers were utilized, a notable enhancement in the flexural strength of URE was recorded, ranging from 60.93% to 70.74% [16]. In another study, Sen and Saha [42] employed coconut coir, bamboo strips, and jute fibers for externally reinforcing small-scale RE wall models. Among these three NF materials, the authors specifically evaluated the flexural capacity of the bamboo-encased RE wallet, which yielded superior results in other mechanical properties such as UCS and shear strength. It was revealed that the flexural strength of the bamboo-encased RE wall increased by 107.06% in comparison to URE. Furthermore, Sen and Saha [42] showed that the flexural strength of NFRE could be enhanced further by employing a double encasement approach (both horizontally and vertically) and treating the NF material with an adhesive agent like bitumen. Bati et al. [127] adopted a different technique to externally attach jute fibers to RE specimens by linking separate RE wall units with a fabric strip. The authors suggested that this method of joining RE blocks resulted in increased flexural strength, although the degree of improvement was not clearly quantified.

In contrast to NFRE, the flexural strength performance of BPRE is typically less explored. Nonetheless, one research investigation [62] reported on the flexural strength of RE stabilized with chitosan biopolymer. This investigation revealed that incorporating chitosan into RE materials at increasing concentrations of 0.5–3.0 wt% could enhance flexural strength by over 300–1160% when compared to URE. Moreover, the samples that included 2.5 wt% and 3.0 wt% chitosan exhibited flexural strengths approximately 11% and 21% greater than CSRE, respectively. It appears that chitosan enhances the flexural strength of RE through a mechanism similar to that of UCS, characterized by the formation of a hydrogel comprising a three-dimensional polymer network within the RE soil matrix, which provides structural support after 7 and 28 days of curing [62].

Concerning the shear strength of RE walls, current standards typically assume a value near or equal to zero if there are no experimental data available [129,130]. A few researchers [16,42,133] have recently worked on enhancing the shear strength of RE walls through bio-based methods. Sen et al. [16] conducted diagonal compression (shear) tests on NFRE wallettes and discovered that incorporating rice straw and jute fibers substantially boosts the peak shearing capacity by 27.39% to 61.90% and 53.42% to 93.33%, respectively, when compared to URE wallettes. Fei et al. [133] explored the combined use of TO, sticky rice slurry, and air lime to improve the shear characteristics (cohesion and internal friction coefficient) of deteriorating RE remains. It was found that the treated RE soil exhibited a cohesion increase of up to 16 times compared to the untreated soil after 180 days of curing, with each additive contributing to overall strength enhancement. However, following two in situ pilot applications, the treatment proved to be only temporarily effective for the duration of one year. Sen and Saha [42] tested the shear strength of NFRE walls and obtained results (0.69–1.46 MPa) that exceeded those recommended in the standards [129,130]. Among the three NFs investigated in their research, bamboo demonstrated the most significant enhancement in shear strength relative to URE (104.61%), followed by jute fiber (44.61%) and coconut coir (36.92%). The notable improvement in strength due to bamboo fibers was attributed to their greater tensile strength in comparison to coir and jute fibers (Table 1). Nonetheless, it is important to mention that Sen and Saha [42] utilized the NF materials solely as confining strips for RE walls. Therefore, it would be interesting to explore how similar materials and various types of NFs might influence the shear behavior of RE when mixed directly with the raw earthen soil. While these three studies alone are insufficient to draw broad conclusions about the ability of biomaterials to enhance the shear strength of RE, they provide a foundation for conducting more comprehensive research in the future.

## 5. Water Durability of BRE

RE walls are susceptible to water-induced damage [134]. An increase in moisture content due to various mechanisms such as capillary water, rainfall, flooding, freeze–thaw, or humidity can lead to a loss of mechanical strength of the RE structure and eventual disintegration over time [135]. Traditionally, methods such as wall coatings, damp-proof courses, and architectural features like roof extensions have been implemented to reduce these issues [135]. However, due to the need to preserve structural esthetics, tackling the moisture vulnerability issue of RE through mix stabilization techniques could be a vital and sufficient alternative approach. In light of this, various researchers have explored the effects of bio-based additives on the water durability performance of RE, examining various aspects, including moisture ingress behavior, hydrophobicity, strength sensitivity in a humid environment, and water-induced erosion, which are discussed in this section. 

### 5.1. Effect on Moisture Ingress Behaviour

In RE structures, rising dampness can occur when water is absorbed by the wall from surface accumulation or the ground, which can eventually jeopardize the stability of the structure. The inclusion of bio-based materials in an RE mix can greatly influence how moisture penetrates, as noted in existing research.

Muguda et al. [136] and Ilman and Balkis [62] conducted contact and suction tests on BRE samples with different biopolymers (XG, GG, and chitosan). They observed how these materials responded to moisture absorption from an external coating (such as mortar or plaster) and capillary rising water from the foundation to the RE wall. At the conclusion of their tests, the authors noted that there were no visible signs of cracking or swelling on the surfaces of the BRE blocks, suggesting their potential to inhibit capillary water penetration into the wall. A more quantitative technique, known as the total water absorption test [130] was utilized by other researchers to assess the water absorption capacity of NFRE [43] and MIRE [17]. Unlike the contact and suction tests, the total absorption test has a definitive pass/fail criterion (for instance, 20%, as noted in [130]). Raavi and Tripura [43] reported a pass result (<20%) for RE bricks mixed with 1% of 25 mm coconut coir fiber and a failure (>20%) when the amount and length of fiber were increased. Mabrouk et al. [46] showed that the presence of HF particles in RE materials increases the capillary absorption capacity and considerably accelerates the absorption kinetics. Porter et al. [17] evaluated the effect of MICP treatment on the water absorption behavior of CSRE blocks. They reported a comparatively higher reduction in water absorption with MICP-treated surfaces (24% reduction) than with pure CSRE blocks (20% reduction).

Fundamentally, three factors influence the penetration behavior of moisture into a building component: the presence of water, a force that transports it, and a porous medium through which it travels [137]. Guihéneuf et al. [53] investigated the capillary absorption kinetics of different BPRE mixtures and characterized their performances based on various parameters that took these factors into account. These parameters include the mass of capillary water (w_cap_), which accounts for the presence of water; the capillary absorption coefficient (A_cap_), which together with the w_cap_ accounts for capillary forces; and a third parameter, A_cap_/n (where ‘n’ is the apparent porosity), which accounts for the porous media involved in the absorption kinetics. Biopolymers such as LO, XG, and vegetable varnish (VV) effectively reduced the capillary water absorption of RE materials. The addition of XG in different amounts showed the most potent capillary reduction (with A_cap_/n values reduced by a factor of 5 to 10), followed by coatings of LO and VV (A_cap_/n reduced by a factor of 2 to 5). On the other hand, bio-additives such as oak seed extract (OSE), Tan, Alg, CG, and casein were comparatively less effective in limiting capillary absorption. They only showed a reduction of A_cap_/n by about 20% compared to samples without additives. It was also observed that a direct correlation exists between the dry density and the water absorption behavior of BPRE mixes. The BPRE mixtures with the lowest dry densities (i.e., OSE, Tan, Alg, CG, and casein) posed limited restrictions on capillary absorption, while those with higher dry densities (LO, XG, and VV) showed a more significant reduction, possibly indicating porosity effects [53].

### 5.2. Effect on Hydrophobicity

The hydrophobicity of an earthen soil is a dynamic surface property that reflects its ability to repel water rather than absorb it and is controlled by many factors such as soil texture (grain size distribution), soil structure (aggregate packing and porosity), and the presence of hydrophobic compounds [122]. It can be quantified using two commonly used parameters: Contact Angle (CA) and Water Droplet Penetration Time (WDPT). CA measurements evaluate the wettability of a material’s surface based on the angle formed between the material surface and a liquid droplet placed on it and are defined by three wetting situations: hydrophilicity (CA = 0), partial wetting (0° < CA ≤ 90°), and hydrophobicity (CA > 90°) [138]. In the WDPT test, a drop of water is applied to the material’s surface, and the time required for the drop to penetrate or be absorbed by the material is measured. A longer penetration time indicates greater hydrophobicity, meaning the material is more resistant to water penetration. Some studies [53,78,122] have applied these test methods to evaluate the hydrophobic effect of biopolymers on RE materials.

Lin et al. [78] examined the hydrophobicity of URE and TO-stabilized RE, finding that raising the TO concentration from 1% to 15% over a drying duration of 28 days resulted in CA and WDPT values reaching 117° and 3600 s, respectively, demonstrating a strong and lasting hydrophobic effect from TO. Given that TO is a drying oil with a high content of unsaturated fatty acids, it can develop a crosslinked network structure in air through oxidative polymerization [80,139]. Lin et al. [78] suggested that this formed crosslinked network structure may contribute to the hydrophobic performance of TO, as it creates a solid or semi-solid coating on the surface of the RE material, which hinders water penetration. Similarly, linseed oil (LO), another lipid-based biopolymer, could potentially enhance the hydrophobicity of RE materials, as demonstrated by significantly elevated CA and WDPT values for Cob Earth in the study conducted by Guihéneuf et al. [53].

### 5.3. Effect on Strength Sensitivity in a Humid Environment

Changes in environmental moisture conditions (humidity and precipitation) can adversely affect the strength and structural stability of RE materials [134,140,141]. Walker et al. [142] indicated that the compressive strength of RE when saturated is likely diminished by about 50% of its dry strength. Heathcote [140] previously noted that a 33–50% decrease in strength for an earthen wall under wet conditions could be deemed adequate performance for strength sensitivity.

Of all the studies referenced in this article, only three—Raavi and Tripura [43], Toufigh and Kianfar [119], and Guihéneuf et al. [53]—investigated the strength sensitivity of BRE. Raavi and Tripura [43] found that coir fiber did not contribute to an improvement in the wet-to-dry compressive strength ratio of URE, falling short of the minimum requirement of at least 0.33 [140]. In the research conducted by Toufigh and Kianfar [119], GG was utilized as a bio-based stabilizer to assess its effect on the strength sensitivity of RE, comparing it with URE and CSRE against the 50% strength reduction limit [140,142]. They determined that the strength loss of GG-stabilized RE with a minor increase in water content was 46%, which indicates acceptable performance; however, URE (39% strength reduction) and CSRE (15% strength reduction) outperformed GG-stabilized RE. Guihéneuf et al. [53] pointed out that XG could lessen the strength sensitivity of RE materials to moisture and further recommended additional research, as XG appears to stop being hydrophilic after reaching a certain moisture content threshold. Conversely, when LO was substituted for XG, Guihéneuf et al. [53] observed no alteration in the strength sensitivity of BRE samples relative to URE, even with raised TO concentrations. Nonetheless, the addition of TO led to a reduction in the moisture content within the BRE mixture, confirming its effectiveness in enhancing hydrophobic properties.

Overall, the above-mentioned studies are neither sufficient nor exhaustive enough to fully understand how different bio-based additives respond to strength variations in wet conditions, which is crucial for engineers to adapt BRE designs to local conditions, ensuring long-term durability.

### 5.4. Effect on Water-Induced Erosion

**Figure 6:**
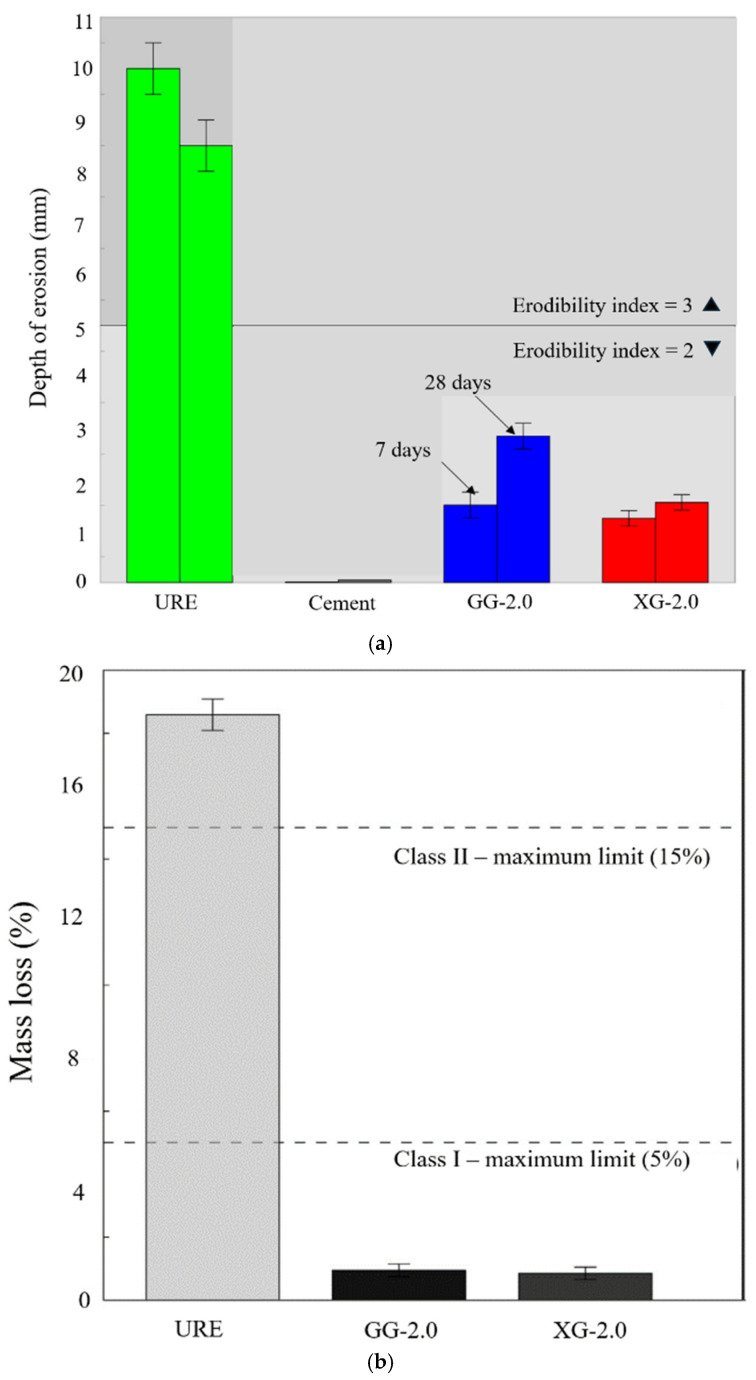

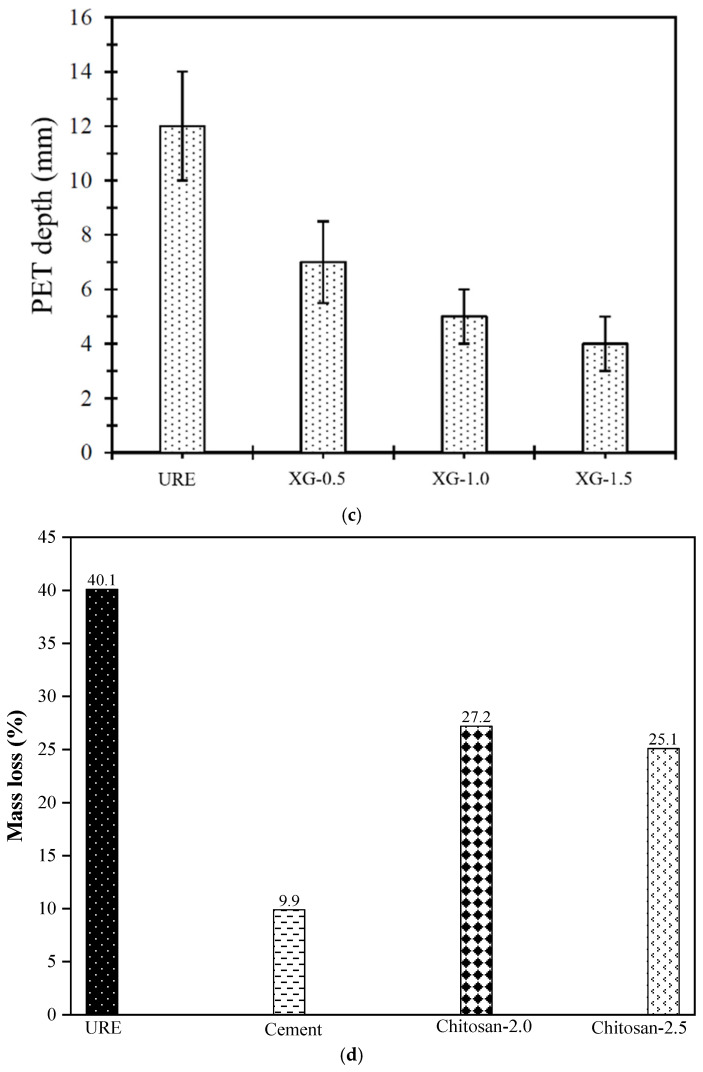

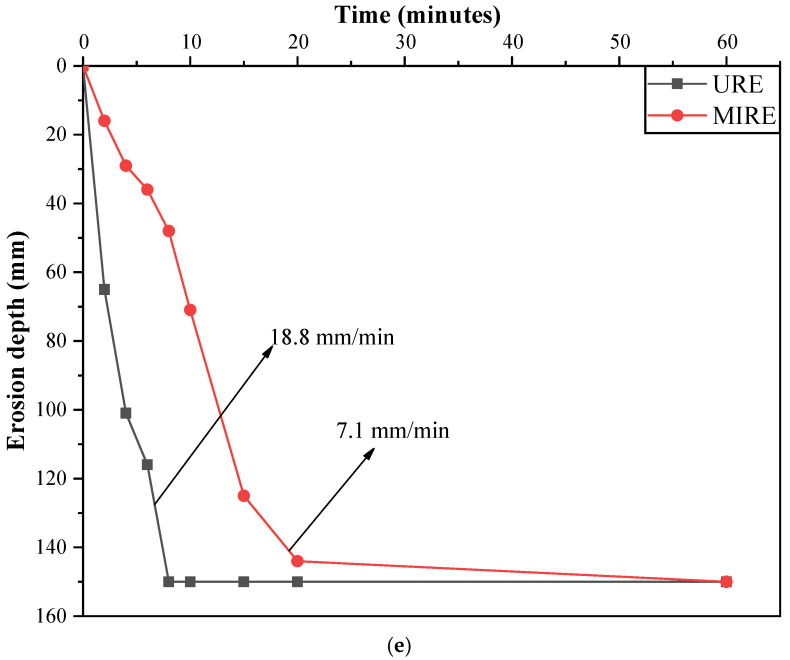
Erosion resistance of different BRE mixtures: (**a**) Geelong drip test results of 7- and 28-day URE, BRE, and CSRE mixtures [136]; (**b**) percentage mass loss of URE and BRE mixtures in the Dip test [136]; (**c**) Pocket Erodometer Test (PET) results of URE and XG-based BRE (data based on [121]); (**d**) percent mass loss of URE, CSRE, and chitosan-based BRE mixtures based on the Immersion test (data based on [62]); (**e**) AET results of URE and MIRE mixtures (data based on [17]).

In real life scenarios, the impact of rainfall on RE walls can be more severe than changes in humidity [143]. Intense wind-driven rainfall during violent storms can lead to considerable erosion of the RE wall surfaces, as the force is adequate to dislodge particles [129]. Generally, overhanging eaves are implemented to reduce erosion [143]. However, during heavy rainfall, more than just these architectural features may be necessary to safeguard the structural integrity of RE walls, placing higher durability demands on the materials themselves [7]. For bio-based stabilizers, it is anticipated that they will take on this challenge, since plain RE soil cannot withstand water damage. Several studies have explored the erosion resistance of various BRE mixtures, including GG, XG, chitosan, and MICP-treated rammed earth. Indeed, a common trend in the literature showed an enhancement in the erosion resistance of BRE mixtures compared to URE, as illustrated in Figure 6.

Muguda et al. [136] performed drip tests to examine the resistance of GG-stabilized and XG-stabilized RE to water erosion. The erosion depths for the samples taken at 7 and 28 days were significantly below the 5 mm threshold established in ref. [144], and the erodability index was 2, indicating satisfactory performance (Figure 6a). Concurrently, the Dip test results indicated a mass loss of less than 5% for both GG- and XG-stabilized specimens (Figure 6b), affirming their appropriateness for use in external wall applications, as per DIN 18,945 [145]. As shown in Figure 6b, XG exhibited a more pronounced effect than GG in reducing the mass loss of the stabilized specimens. Patwa et al. [121] also noted an enhancement in the water erosion resistance of RE due to XG, whereby PET results demonstrated a decrease in erosion depths from approximately 12 mm for URE to 4 mm for specimens containing 1.5 wt% XG (Figure 6c). Chitosan, another type of biopolymer, was examined by Ilman and Balkis [62] for its effectiveness in diminishing surface erosion in RE materials. The erosion performance of RE stabilized with chitosan, as shown in Figure 6d, was improved compared to URE. Nonetheless, none of the samples from this study achieved the <5% mass loss criterion outlined in DIN 18,945 [145]. Lastly, Porter et al. [17] explored the impact of MICP on the erosion resistance of RE materials, utilizing a more rigorous testing approach, i.e., the Accelerated Erosion Test (AET). The findings are presented in Figure 6e, revealing that the MIRE mix reached full penetration in 21.1 min with an erosion rate of 7.1 mm/min, in contrast to the URE samples, which achieved this in 8.0 min at a rate of 18.8 mm/min, indicating enhanced erosion resistance attributable to the MICP treatment.

Table 4 encapsulates the key findings related to the water durability evaluation of BRE across various discussed topics, detailing the bio-additive types and amounts, sample curing durations, and testing methods employed by different authors.

## 6. Discussion on Current Challenges and Perspectives for Future Research

Although BRE technology shows considerable potential in mechanical and water durability performance, certain significant challenges could hinder its broader implementation in industrial settings. This section addresses these challenges and offers important perspectives for future research.

### 6.1. Hydrophilic and Biodegradable Nature of Bio-Based Materials

As demonstrated through experience, many bio-based materials inherently tend to absorb water (hydrophilic) and are vulnerable to biological degradation (biodegradable). These characteristics are not advantageous when applied to rammed earth, as they ultimately result in reduced strength and a shorter service life for the BRE structure. The absorption of moisture can lead to dimensional changes in the natural fiber or biopolymer, thereby impacting the interfacial adhesion and mechanical properties of the BRE composite. At the same time, biodegradation over time (which can occur within hours to years, influenced by various factors such as microorganisms, pH, temperature, sunlight, and humidity [146]) can weaken the bond strength of the polymer monomer units [147] and diminish their reinforcing effects on RE materials.

To counter these problems, some researchers have indicated that pre-treatments may be required for NFs before they are mixed with earth [24,42], whereas for biopolymers, crosslinking could enhance hydrophobic properties and improve resistance to degradation by promoting the better interlocking of monomers [60]. A diverse array of pre-treatment techniques is available for NFs, including sodium chlorite, methacrylate, silane, peroxide, acetylation, bitumen, enzyme, plasma, and ozone treatment, which can enhance functionality against microorganisms and boost hydrophobicity [148]. Likewise, various crosslinking methodologies can be utilized depending on the biopolymer’s nature, which encompass physical methods (such as ionic interactions, crystallization, stereo-complex formation, hydrophobized polysaccharides, protein interactions, and hydrogen bonding) and chemical techniques (like irradiation, sulfur vulcanization, or chemical reactions) [149]. However, despite the established effectiveness of these methods, they have not been extensively explored for RE applications. In all the literature reviewed in this study, only two studies were identified that employed either pre-treated fibers or crosslinked biopolymers for RE stabilization. Sen and Saha [42] utilized bitumen to pre-treat jute, coconut coir, and bamboo fibers prior to their use as external reinforcement for model RE wall units. Muguda et al. [60] demonstrated that crosslinking XG with another biopolymer (GG) led to the generation of hydrogels with improved physical integrity and mechanical properties when applied in RE stabilization. While further research in this area is advisable (considering that pre-treatment and crosslinking also enhance the strength and durability of the RE mix), the potential increase in material costs and possible adverse environmental impacts due to pre-treatment substances and crosslinking agents should be taken into account. Therefore, future research tackling the challenges of biodegradation and hydrophilicity of biomaterials in RE applications must consider practical contexts. Choosing an appropriate pre-treatment or crosslinking method and material should strive for a reasonable balance between structural performance, cost, and environmental sustainability.

### 6.2. Soil Variability Issues

Unlike manufactured materials such as steel, aluminum, and concrete, natural soil deposits present variability challenges. Even if soils share similar sedimentation histories and environments, their geotechnical properties—such as granularity, mineral composition, voids, specific gravity, in situ density, void ratio, moisture content, and Atterberg limits—can differ significantly [143], impacting their potential for effective stabilization [150]. In practical scenarios, local engineers are often faced with diverse soil types from a site, necessitating a decision on the most appropriate option for bio-stabilization. While optimal performance with BRE mixtures is partly influenced by the properties of the bio-based additive, the characteristics of the substrate soil are also crucial. The studies examined in this paper based their selection of the substrate soil on adherence to the particle size distribution (PSD) curves recommended for URE mixtures by multiple guidelines [129,130,144]. However, past research [150,151] has shown that for stabilized rammed earth (SRE), the soil’s PSD alone cannot determine its stabilization suitability, and they proposed alternative distinguishing properties. Burroughs [150] performed an extensive study involving 104 different soils treated with cement and lime, demonstrating that linear shrinkage and plasticity index properties are better indicators than PSD for assessing soil suitability for SRE, based on the criterion of a 2 MPa compressive strength. In contrast, Ciancio et al. [151] favored the drying shrinkage property over linear shrinkage and asserted that guidelines related to the plasticity and PSD of the substrate soil should not solely focus on strength considerations but should also take durability into account. Although these studies were limited to the chemical-based stabilization of rammed earth (CSRE), they highlight the necessity for establishing distinct soil selection criteria for URE and SRE mixtures. Consequently, future research should explore whether the suggested soil selection criteria for CSRE can be applied to BRE mixtures or if new criteria should be developed for this specific design purpose.

### 6.3. Limited Research on the Mechanical Performance of BRE

Most of the existing studies on the mechanical performance of BRE have concentrated on compressive strength, while other properties such as shear, tensile, and flexural strength have received relatively limited attention. While compressive strength tends to be emphasized in the design of RE materials, further exploration of the aforementioned parameters is also crucial for achieving a well-rounded understanding of the mechanical characteristics of BRE materials, thus enhancing reliability and encouraging broader industrial adoption. Moreover, the evaluation of compressive strength shows that there are inconsistencies in standard testing protocols, which is an issue that needs to be thoroughly addressed. Indeed, it can be argued that variations in specimen shapes, loading conditions, and data processing techniques specified in ASTM standards, European standards, or other improvised in situ UCS measurement methods may result in discrepancies in the recorded values of strength and elastic modulus. Consequently, it is essential to critically assess the testing methods and specifications to determine their relevance to BRE mixtures. Furthermore, a comprehensive framework for UCS testing can be established by meticulously designing a test program that explores factors such as specimen geometry (including shape, dimensions, height-to-diameter ratios, and constraints on mold sizes), loading rates, edge preparation and tolerances, curing conditions, and methods for evaluating precision and bias within the RE context. Another potential avenue for future research could involve rigorously defining an empirical correlation between the tensile and compressive strength of BRE mixtures, as has been determined for URE [47,128]. Additionally, a comprehensive study of how saturation and density influence the tensile, shear, and flexural properties of BRE is necessary, which may contribute to advancing the overall design and construction practices of BRE materials in seismically active areas.

### 6.4. Issues Related to Water Durability Assessment Methods

In general, the water durability assessment of BRE has predominantly relied on laboratory techniques, which are often seen as impractical due to their limited correlation with actual long-term exposure conditions [152,153]. At their best, these methods only offer conservative estimates that more accurately depict short-term durability [7]. To enhance reliability, certain modifications to the laboratory testing procedures and the interpretation of results are considered essential [152]. For example, in erosion evaluations, critical rainfall parameters such as intensity, drop size, impact velocity, kinetic energy, and angle of impact could be adjusted to better represent the natural rainfall typical of the specific study area, and the analysis of erosion depth or weight loss results should be based on actual field events. Furthermore, performing laboratory tests alongside long-term in situ tests would yield more dependable data regarding the durability of BRE walls, since all climate and time-dependent factors would be integrated. Unfortunately, the literature still lacks quantitative in situ measurement methods for RE walls.

In situ techniques are more appropriate when they are user-friendly and quick to execute, while also yielding valuable information for evaluating in-service performance. Bui et al. [154] proposed stereo-photogrammetry as an in situ assessment method to quantify erosion on URE walls subjected to 20 years of natural weathering. Other non-destructive testing (NDT) methods, including Infrared Thermography (IRT), electrical resistance measurements (ERM), and in situ dynamic assessments utilizing accelerometer and velocimeter sensors [155], also have the capability to quantify and evaluate the long-term durability of RE materials in the field. Nevertheless, these techniques face challenges such as requiring a high level of technical expertise, significant initial investment in equipment and software, intricate data processing, and dependence on climatic conditions [135]. Regardless, their ability to deliver precise and dependable assessments of the long-term durability of RE walls in situ could assist in calibrating and refining more relevant laboratory testing methodologies and effective performance prediction models. Indeed, creating and utilizing long-term performance prediction models based on actual data would serve as a valuable resource for evaluating the engineering sustainability of BRE technology. According to Morel et al. [135], the surface erosion process of RE walls over time does not adhere to a linear model. At first, surface erosion occurs at a faster rate, but as time progresses, the erosion tends to stabilize. Any future performance prediction model must consider this non-linear trend, along with the erosion mechanisms and their relationship to the reduction in compaction energy caused by friction against the formwork (since the soil in contact with the formwork tends to be less compacted and thus erodes more quickly) [135].

## 7. Summary and Conclusions

The use of bio-based materials in rammed earth construction is gaining more significance as it promotes a shift towards building methods that utilize lower energy and emissions-intensive materials, enhancing efficiency. This study offers a comprehensive review of the stabilization of RE with bio-based agents, concentrating on how these materials impact key mechanical properties, including UCS, tensile strength, the modulus of elasticity, shear strength, and flexural strength, along with their performance in water durability, which encompasses erosion resistance, moisture ingress behavior, strength sensitivity, and hydrophobicity. A total of 45 studies were reviewed, with the majority focusing on NFs (50% of the total documents reviewed), followed by biopolymers (37%) and bio-cement (13%). Based on the findings of this study, the following main conclusions can be drawn:

1. The compressive strength of RE is generally improved using biopolymers, with most studies showing an increase of around 13.3% to more than 100% compared to URE. Biopolymers enhance strength through surface coating, interparticle binding, or void filling.
2. Natural fibers (NFs) and biocementation through MICP treatment have the potential to enhance the UCS of RE materials; however, the effectiveness is contingent upon several variables. For NFs, it is advisable to limit usage to no more than 1 wt% and a fiber length of 25 mm for stabilizing RE. Also, a fiber type that possesses high modulus, high tensile strength, and low water absorption typically benefits UCS enhancement. Regarding MICP, the UCS increase is reliant on the concentrations of both microorganisms and calcium present in the intergranular medium, as well as the nitrogen source.
3. The reported tensile strengths of different BRE mixes appeared to be generally low, ranging from 0.1 to 0.5 MPa. However, their corresponding tensile-to-compressive (T/C) ratios fall between 0.07 and 0.11, which is considered acceptable for RE materials.
4. The literature reveals a broader dispersion in the elastic modulus of BRE mixtures, with values spanning from 29.2 to 511 MPa. This variability arises from differing calculation methods employed by various authors, challenges in measuring strain, and variations in the bio-additive materials utilized. This study emphasizes the importance of establishing reliable standard procedures for strain measurement and the mathematical calculation of the elastic modulus of BRE mixtures.
5. Some studies indicate that using biopolymers can improve RE’s water durability by limiting moisture ingress, reducing strength sensitivity in a humid environment, and improving erosion resistance. Furthermore, lipid-based biopolymers can increase hydrophobic properties by coating RE materials’ surfaces, thereby decreasing water penetration.
6. The surface treatment of RE materials by MICP helps to reduce the erosion rate and limits water absorption to a certain extent. However, the durability of NFRE still needs to be thoroughly investigated.
7. Holistically, the existing body of literature on BRE technology still needs improvement. By delving deeper into the possibilities of novel bio-based materials, improving the performance of current biopolymers and NFs through crosslinking and surface treatment, respectively, establishing suitable criteria for soil selection in BRE, broadening the assessment of BRE’s mechanical performance to encompass other pertinent parameters discussed in this study, and improving the reliability of BRE’s water durability assessment through a combination of laboratory and in situ long-term performance data, researchers can help shape this healthy, more sustainable and resilient building practice for generations to come.

While the scope of the present study was limited to the impacts of bio-based treatments on the mechanical performance and durability of RE mixtures, it is recommended that future research give adequate consideration to other operational performance characteristics such as hygrothermal behavior, acoustic performance, and thermal comfort. Furthermore, a comprehensive Life Cycle Analysis should be conducted on the various types of BRE mixtures discussed in this study to assess their cost performance, energy consumption, and carbon emission in comparison to the traditional chemically stabilized rammed earth.

## Figures and Tables

**Table 1:** Physical and mechanical properties of natural fibers used in rammed earth.

NF Material	Fiber Amount (wt% of Dry Soil)	Physical Properties							Mechanical Properties		Ref.
		Length (mm)	Thickness (mm)	Width (mm)	Diameter (mm)	Density (g/cm^3^)	Ultimate Elongation (%)	Water Absorption (%)	Tensile Strength	Elastic Modulus (GPa)	
Coconut coir	N/A	-	0.9	-	-	0.98	1.08	-	28	20.4	[42]
	1, 3, 5	25, 50	-	-	0.4	1.27	-	-	80	-	[43]
	0.5	50	-	-	-	-	-	-	83.23	-	[44]
Hemp	0.75, 1.25	-	0.13	0.65	0.41	1.4	-	-	276	21.7	[45]
	25%*v*/*v*	-	-	-	-	0.105	-	345	-	-	[46]
Jute	N/A	-	0.85	-	-	1.23	1.46	-	35	24.5	[42]
Bamboo	N/A	-	0.85	10, 15	-	1.12	1.77, 1.89	-	49.5, 51.7	19.7	[42]
Barely straw	0.75	20–30	-	-	1–3	0.72	-	175	6–12	0.3–0.6	[47]
Date palm	0.75	30	-	-	1–2	0.94	-	89	233	5	[47,48]
Rice straw	N/A	-	-	-	-	0.98	2.26	-	14.76	20.8	[16]
Wheat straw	0.25	30	-	-	-	-	-	280–300	22–79	5–6	[49,50]
Sheep wool	0.25	Up to 100	-	-	-	-	-	-	-	-	[15]

N/A = not available, %*v*/*v* = percent by volume of dry soil.

**Table 2:** MICP application for RE stabilization.

Bacterial Strain	Nutrient Delivery Method	Urea Concentration (g/L)	Cementation Solution Composition	Application Rate Cementation Solution During RE Treatment	Ref.
*Bacillus subtilis*, *S. pasteurii*, and *Bacillus subtilis* subsp. *Subtilis*	Mixing method	20	50 mL	-	[99]
*S. pasteurii*	Mixing method	20	500 mM urea + 500 mM CaCl_2_	250 mL in 8 kg of dry soil	[103]
*S. pasteurii*	Mixing method	20	500 mM urea + 500 mM CaCl_2_	-	[19]
*S. pasteurii*	Gravimetric injection method	-	500 mM urea + 500 mM CaCl_2_	17 mL sprayed on the RE surface for 24 h followed by 8 mL sprayed twice a day for 24 days	[17]
*Bacillus megaterium*, *Bacillus sphaericus*, and *Bacillus* sp.	Mixing method	-	2% urea solution + 25 mM CaCl_2_	100 mL applied in RE mix	[112]

**Table 3:** Summary of experimental results on mechanical strength parameters for different BRE mixes.

Bio-Based Additive		Optimum Additive Amount (wt%)	Compressive Mechanical Parameters				Tensile Mechanical Parameters		Tensile-to-Compressive (T/C) Strength Ratio ^2^	Ref.
			UCS (CoV) (MPa)	Peak Strain (CoV) (mm/mm)	Elastic Modulus (CoV) (MPa)	Compressive Strength Improvement (%) ^1^	Tensile Strength (CoV) (MPa)	Tensile Strength Improvement (%) ^1^		
Natural fiber	DP	0.75	3.25 (4%)	0.0172 (3%)	189 (1%)	↑ 62.5	0.46	↑ 12.5	0.14	[47]
		1.0	2.29 (4.5%)	0.0183 (10.8%)	454 (9.7%)	↑ 29.4	0.19 (3.1%)	↑ 9	0.08	[48]
	BS	0.75	2.74 (7%)	0.0194 (7%)	141 (2%)	↑ 37	0.50	↑ 25	0.18	[47]
	WS	0.25	2.58 (3.1%)	0.036	71 (6.32%)	-	0.24 (11.7%)	-	0.09	[49]
		0.5	0.76	0.0129	126	↑ 33.3	0.22	↑ 29.4	0.29	[50]
	SWF	0.25	2.67 (6.7%)	-	194 (19.6%)	↑ 6.1	-	-	-	[15]
	CIT	1.0	1.90 (5.8%)	-	197 (11.7%)	↓ 24.5	-	-	-	[15]
	GRA	1.0	2.14 (26.6%)	-	250 (22.8%)	↓ 14.7	-	-	-	[15]
	Coconut coir	1.0	4.40 (6.4%)	-	-	↑ 15.2	0.25 (12%)	↑ 78.6	0.06	[43]
	HF	1.25	-	-	-	-	0.48 (29.1%)	↑ >100	-	[45]
		25 vol%	1.04	0.0497	29.2	↓ 43	-	-	-	[46]
	Jute fabric	-	2.24 (9.8%)	-	34.6 (55.1%)	↑ 75	-	-	-	[127]
	Sisal	1.0	5.26		254	↑ 33.1				[117]
Biopolymer	GG	2.5	2.64 (0.8%)	0.0216	131 (2.8%)	↑ >100	0.27 (21.1%)	↑ 12.5	0.10	[119]
		3.0	4.40	0.0225	-	↑ >100	0.125	↑ >100	0.03	[116]
	XG	3.0	4.25	0.0195	-	↑ >100	0.288	↑ >100	0.07	[120]
		1.5	5.35	0.0175	380	↑ >100	-	-	-	[121]
		1.0	5.58	-	-	↑ >100	-	-	-	[71,72]
	LIG	1.0	3.47 (17%)	-	511 (10.4%)	↑ 38.3	-	-	-	[15]
	TAN	1.0	2.85 (8.8%)	-	270 (21.9%)	↑ 13.3	-	-	-	[15]
	Animal glue	1.0	6.86	-	-	↑ >100	-	-	-	[71,72]
	TO	10	2.64	-	-	↑ >100	-	-	-	[78]
	BP	73.8 mL/kg	6.71	-	-	↑ 36	-	-	-	[70]
MICP	*S. pasteurii* bacteria	-	0.6 (14-day strength)	-	-	↑ >100	-	-	-	[70]
	Urea + *S. pasteurii*	250 mL in 8 kg of dry soil	3.3	-	-	↓ 31.4	-	-	-	[103]
		17 mL sprayed on the RE surface	0.8	0.029	105	↑ 33.3	-	-	-	[17]
	Blood + *S. pasteurii*	250 mL in 8 kg of dry soil	6.21	-	-	↑ 45	-	-	-	[103]

Notes: All results are average values of 28-day cured BRE mixes. ^1^ Percentage improvement relative to URE. ^2^ Recommended T/C ratio by Bui et al. [128] = 0.07–0.11. ↑ means increase, ↓ means decrease, ↑ > means increase by more than, and - means that the results are not reported.

**Table 4:** Summary results of water durability performance of different BRE mixes.

Water Durability Assessment Topic	Bio-Based Treatment	Optimum Bio-Additive Amount (wt%)	Curing Time of BRE Samples (Days)	Durability Test Method (Test Parameter)	Main Finding (s)	Ref.
Moisture ingress behavior	Coconut coir fiber	1.0	28	Total absorption test (% water absorption)	RE blocks with 1 wt% of 25 mm coir passed the water absorption requirement of <20%, as per Walker [130], but failed as the fiber amount and length increased	[43]
	MICP	-	28	Total absorption test (% water absorption)	MICP-treated samples achieved a 24% lower water absorption than untreated samples	[17]
	Chitosan	2.5	28	Contact test (visual cracks and swelling) Suction test (visual cracks and swelling) Immersion test (% mass loss)	No visible signs of cracks or swelling were observed on the surface of the BRE blocks	[62]
	GG, XG	2.0	28	Contact test (visual cracks and swelling) Suction test (visual cracks and swelling)	No visible signs of cracks or swelling were observed on the surface of the BRE blocks	[136]
	ALG, XG, CG, LO, casein, CT, OSE, VV	0.5–2.0	7	Capillary water absorption (CWA) test (Acap/n)	XG, LO, and VV reduced the Acap/n by a factor of 2–10, while ALG, CG, casein, CT, and OSE showed less reduction effect XG showed the highest restriction in capillary absorption, reducing the Acap/n by a factor of 5–10	[53]
Hydrophobicity	TO	15.0	3, 7 and 28	Sessile drop contact angle (SDCA) test Water drop penetration time (WDPT) test	TO induced high and persistent hydrophobicity in the RE soil matrix in a time-dependent manner. As TO concentration increases up to 15 wt%, SDCA and WDPT values reach 117o and 3600 s, respectively	[78]
Strength sensitivity in a humid environment	GG	2.5	28	Strength testing	The compressive strength of BRE samples was reduced by 46% under wet conditions, indicating satisfactory performance as per test requirement (i.e., <50% reduction)	[119]
Water-induced erosion	MICP	-	28	AET (erosion rate)	MICP treatment improved the erosion performance of RE, as MIRE specimens reached full penetration depth in 21.1 min at an erosion rate of 7.1 mm/min compared to URE’s 8.0 min at 18.8 mm/min	[17]
	XG	1.5	7	PET (erosion depth)	XG significantly reduced the PET erosion depth, going from 12 mm for URE to about 4 mm for samples mixed with 1.5 wt% XG	[121]
	GG, XG	2.0	7 and 28	Dip test (% mass loss) Geelong drip test (erosion depth)	GG and XG effected a mass loss of <5% and erosion depth of <5 mm, satisfying DIN 18,945 [1] requirement for naturally exposed external wall and Frenchman’s recommendation [2], respectively Biopolymer specimens with GG erode faster with increased curing age, while those with XG were unaffected	[136]
	Chitosan	2.5	28	Immersion test (% mass loss)	Mass loss > 5%, failing to meet the DIN 18,945 [1] requirement for naturally exposed external wall	[62]
